# The efficacy of periarticular drug infiltration for postoperative pain after total hip arthroplasty: A systematic review and meta-analysis

**DOI:** 10.1097/MD.0000000000006401

**Published:** 2017-03-24

**Authors:** Yanyang Wang, Fuqiang Gao, Wei Sun, Bailiang Wang, Wanshou Guo, Zirong Li

**Affiliations:** a The Graduate School of Peking Union Medical College; b Centre for Osteonecrosis and Joint-Preserving & Reconstruction, Department of Orthopedic Surgery, Beijing Key Laboratory of Arthritic and Rheumatic Diseases, China-Japan Friendship Hospital, National Health and Family Planning Commission of the People's Republic of China, Beijing, China.

**Keywords:** efficacy and safety, hip arthroplasty, intraoperative periarticular drug infiltration

## Abstract

**Background:**

The ability of intraoperative periarticular drug infiltration (PDI) to control pain after total hip arthroplasty (THA) has been studied for many times, but it still remains controversial. Therefore, we undertook a meta-analysis to evaluate the efficacy and safety of PDI on postoperative pain after THA.

**Methods:**

Databases, including Pubmed, Medline, Embase, Web of Science, and Cochrane library, were searched to identify randomized controlled trials concerning PDI for pain management in patients undergoing THA. The primary outcomes included pain score with rest or activity and opioid consumption. Secondary outcomes were length of hospital stay and complications (nausea or vomiting).

**Results:**

A total of 666 THA patients from 8 randomized controlled trials were subjected to meta-analysis. The results showed that the PDI group had better pain relief, less opioid consumption, and less length of hospital, when compared with the placebo group (*P* < 0.05). No significant differences were observed in regard to visual analog score with activity and complications between the 2 groups.

**Conclusion:**

PDI may be recommended for the pain management after THA. However, due to the variations in the included studies, additional studies are still needed to validate these conclusions.

## 1 Introduction

For most patients with degenerative joint diseases of the hip such as osteoarthritis, total hip arthroplasty (THA) is a preferred option.^[1]^ However, THA can be associated with moderate to severe pain in the early postoperative period.^[2]^ Most of us focused on how to control the postoperative pain since we considered that the moderate to severe pain^[3,4]^ was the true reason why patients need to stay in the hospital for a longer time and might also have more complications, then many kinds of analgesics emerge, like epidural analgesia (EA), peripheral nerve blocks (PNBs), and patient-controlled opioid administration,^[5,6]^ which were introduced to the operating room. As these techniques are widely used, local and systemic side effects like urinary retention and pruritus and spinal cord ischemia are found. An ideal analgesia regimen in THA should preserve knee mobilization ability, enable earlier physical therapy, hasten recovery, shorten hospital stay, lower the risk of postoperative complications, and improve patient satisfaction.

In recent years, periarticular drug infiltration (PDI) after THA was developed and analyzed to assess the efficacy of this method to control pain. According to the meta-analysis by Jiang et al,^[7]^ periarticular multimodal drug injection should be recommended for the pain management after total knee arthroplasty (TKA) or THA, and they found that PDI group had better pain relief, less opioid consumption, larger range of motion, and lower rates of nausea and vomiting than the placebo group; no significant difference was seen in regard to the length of hospital stay between the 2 groups. However, some studies^[8–10]^ reported that drug infiltration could result in decreased pain. Murphy et al^[8]^ and Busch et al^[9]^ also reported that it could reduce postoperative patient-controlled analgesia requirements. However, all the other studies^[9–16]^ reported that there was no significant difference between the local infiltration group and control group in this aspect. Whether PDI offers superior analgesia and faster early postoperative recovery after THA remains controversial. Given this, we performed this meta-analysis to assess the clinical efficacy and safety of PDI for perioperative pain management after THA.

## 2 Materials and methods

This meta-analysis was carried out in accordance with the Preferred Reporting Items for Systematic Reviews and Meta-Analyses reporting guidelines for the meta-analysis of intervention trials.^[17]^ Ethical approval for this study was unnecessary because it was a review of existing literature and did not involve any handling of individual patient data.

### 2.1 Search strategy

PubMed, Medline, Embase, Web of Science, and the Cochrane Library were searched up to March 2016 for comparative studies involving PDI in the management of pain relief after THA. The search terms included “local infiltration” OR “periarticular injection” OR “hip arthroplasty” OR “hip replacement” OR “joint prosthesis” OR “joint arthroplasty” AND “RCT.” We added Medical Subject Headings in all searches as long as it was available. Additionally, we searched the reference lists of relevant reviews and included studies to identify potentially eligible studies. Two authors conducted all the searches independently without publication status and language restrictions. Differences were resolved by discussion.

### 2.2 Inclusion criteria and study selection

We identified randomized controlled trials (RCTs) (by) comparing with control group. The outcome measure included pain score by visual analog score (VAS) with rest or activity, analgesic consumption, range of motion, length of hospital stay, and complication (nausea or vomiting, infection, wound complication, blood loss). Articles that reported at least 1 outcome was included and those without the outcome measures of interest were excluded. Quasi-RCT or non-RCT, retrospective studies, letters, comments, editorials, and practice guidelines were excluded. The potentially relevant citations for inclusion were assessed by 2 authors together.

### 2.3 Data abstraction and quality assessment

Two authors independently reviewed all titles and abstracts of studies identified by the above searches. Full texts of any potentially useful studies were reviewed, and disagreements were resolved by discussion. Data on patient characteristics (age, sex, and other baseline characteristics), intervention, and outcomes were extracted in duplicate by the 2 authors, using a standardized form. The postoperative pain intensity was measured by 10 points VAS. When a numerical rating scale (NRS) score was used, it was converted to a VAS score. Data in other forms (ie, median, interquartile range, and mean ± 95% confidence interval [CI]) were converted to mean ± standard deviation (SD) according to Cochrane Handbook.^[18]^ If the data were not reported numerically, we extracted them by manual measurements from the published figures.

Two authors independently assessed the risk of bias of the included studies based on the following items: random sequence generation, allocation concealment, blinding, incomplete outcome data, selective outcome reporting, and other sources of bias.^[18]^ Disagreement was resolved by the third author. The quality of evidence of outcomes was judged according to the Grading of Recommendations Assessment, Development, and Evaluation (GRADE)^[19]^ criteria. Two authors independently evaluated 5 factors (risk of bias, inconsistency, indirectness, imprecision, and publication bias) that may downgrade the quality level of evidence. The recommendation level of evidence was classified into 4 categories: high, moderate, low, or very low.^[19]^ High quality: further research is very unlikely to change our confidence in the estimate of effect; moderate quality: further research is likely to have an important impact on our confidence in the estimate of effect and may change the estimate; low quality: further research is very likely to have an important impact on our confidence in the estimate of effect and is likely to change the estimate; very low quality: we are very uncertain about the estimate.

### 2.4 Statistical analysis

All calculations were performed through RevMan v5.3 software. Mean difference (MD) with 95% CI was calculated for the continuous data and odds ratios (ORs) with 95% CI for the dichotomous data. Heterogeneity among studies was estimated with I^2^ statistic, and substantial heterogeneity was represented by an I^2^ > 50%. A fixed-effects model was used if the heterogeneity test did not reveal statistical significance (I^2^ < 50%, *P* > 0.1). Otherwise, we adopted the random-effects model. *P* < 0.05 was considered to be statistically significant. Sensitivity analysis was performed to explore the impact of an individual study by deleting 1 study each time.

## 3 Results

### 3.1 Search results

**Figure 1:**
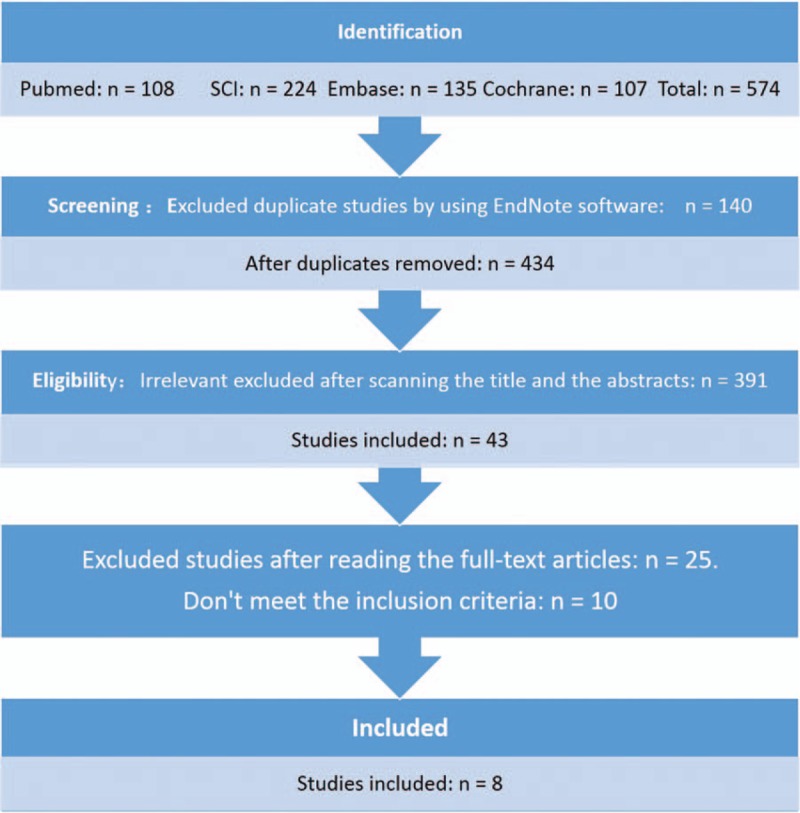
The flow chart of literature screening.

We found 574 citations after comprehensive searches. Then, we excluded 140 duplicates by using EndNote software. After screening the titles and abstracts, 391 studies which were not relevant to our analysis were excluded. There were 35 citations that did not fulfill inclusion criteria excluded after reading full texts. Eight RCTs^[8–14,16]^ were identified in our study at last (Fig. 1).

### 3.2 Characteristics of included studies

In all, 666 THA participants in 8 RCTs were involved. The medicines of local infiltration analgesia in these 8 RCTs include ropivacaine, bupivacaine, morphine, ketorolac, and epinephrine; one kind of them or a mixture of several kinds of the drugs were injected into the soft tissues around the surgical area. The control group got infiltration of equal volume of saline without medicine or did not get any infiltration. Table 1 shows the baseline characteristics of the 2 groups.

**Table 1:**
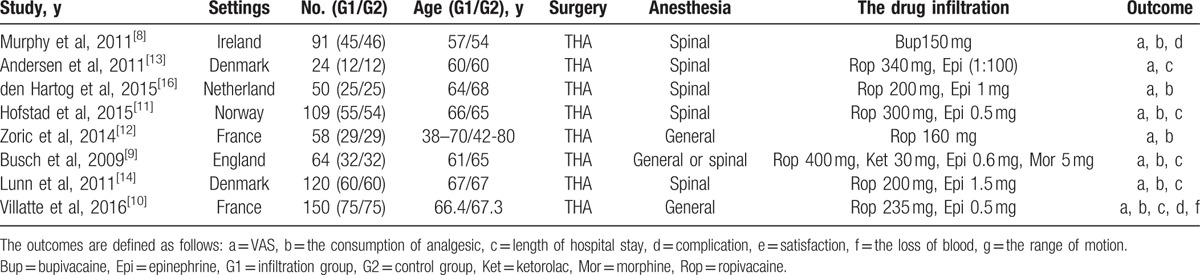
Characteristics of included studies.

### 3.3 Quality of the evidence by GRADE system

Table 2 shows the risks of bias in the included studies and the evidence GRADE.^[19]^ Among the included studies, 4 were randomized by randomization table and 4 by computer-generated numbers. Four studies reported allocation concealment using sealed envelope or box, whereas the other 4 were unclear. Among all the studies, participants and outcome assessors were double-blind. We used the GRADE^[19]^ criteria to measure the strength of recommendations. The evidence quality for each outcome was mostly moderate or low. GRADE^[19]^ system was used to evaluate all outcomes in this meta-analysis. The evidence quality for each outcome was low or moderate or high. We listed the results of the evidence of outcomes in Table 3.

**Table 2:**
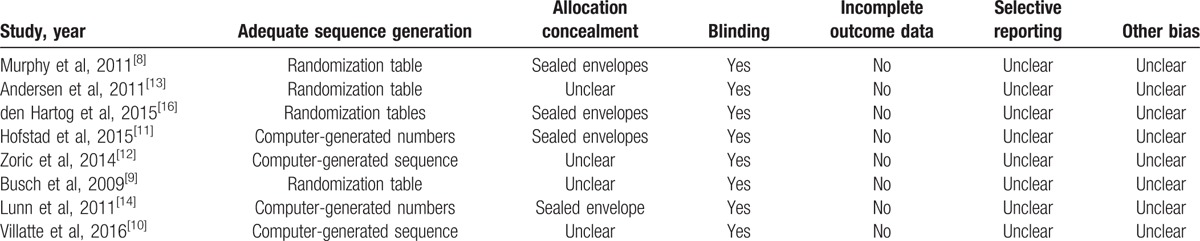
Risk of bias in included studies.

**Table 3:**
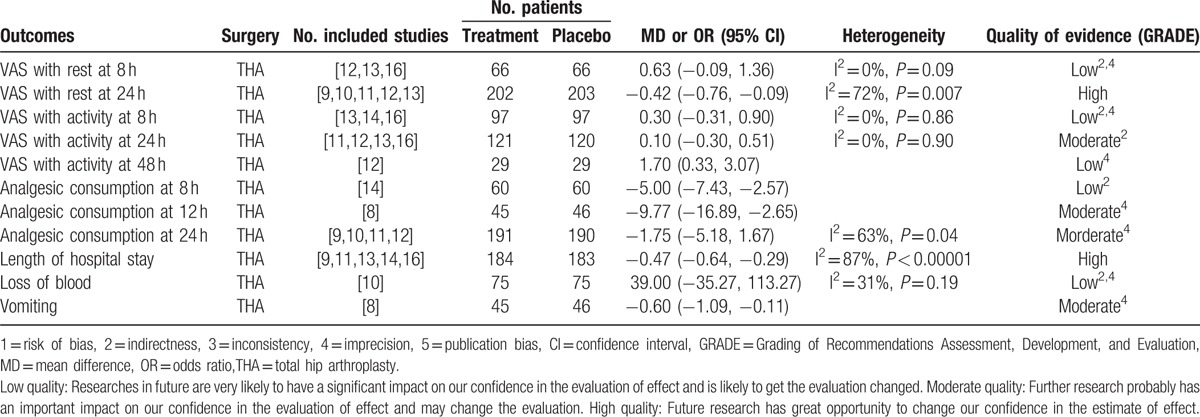
The quality of each outcome for THA using GRADE system.

### 3.4 Results of the meta-analysis

#### 3.4.1 VAS score with rest

Meta-analysis of 3 studies^[12,13,16]^ with 132 patients after THA showed that there was no significant difference between the PDI group and control group at 8 hours (MD 0.63, 95% CI −0.09 to 1.36, *P* = 0.09; Table 3) postoperatively. But meta-analysis of 5 studies^[9–13]^ with 405 patients showed that the PDI group had lower VAS score at 24 hours (MD −0.42, 95% CI −0.76 to −0.09, *P* = 0.007; Table 3) postoperatively.

#### 3.4.2 VAS score with activity

Meta-analysis of 5 studies^[11–14,16]^ with 361 patients after THA demonstrated that there was no significant difference of VAS score with activity between the PDI group and control group at 8 hours (MD 0.30, 95% CI −0.31 to 0.90, *P* = 0.86; Table 3), 24 hours (MD 0.10, 95% CI −0.30 to 0.51, *P* = 0.90; Table 3), and 48 hours postoperatively.

#### 3.4.3 Analgesic consumption

Meta-analysis of 4 studies^[9–12]^ with 381 patients after THA showed that the PDI group had less analgesic consumption at 24 hours (MD −1.75, 95% CI −5.18 to 1.67, *P* = 0.04; Table 3) than the control group.

#### 3.4.4 Length of hospital stay

Meta-analysis of 5 studies^[9,11,13,14,16]^ with 367 patients after THA showed that the PDI group had less length of hospital stay (MD −0.47, 95% CI −0.64 to −0.29, *P* < 0.00001; Table 3) than the control group.

#### 3.4.5 Complications

One study^[9]^ reported the data of wound infection in patients after THA. In this study, 3 patients had a minor wound problem in the PDI group and 1 in the control group. However, these included 3 patients with blisters related to their dressings and 1 prominent suture requiring trimming. One patient in the PDI group had a deep vein thrombosis postoperatively. Four studies^[10,12,13,16]^ had not found the complications of infection, wound complication, and nausea, and just reported that there was no significant difference between the 2 groups about the complications. One study^[10]^ also reported the loss of blood between the 2 groups, but no obvious difference was found.

## 4 Discussion

The results of this meta-analysis provided moderate evidence that the PDI group, compared with the control group, just had better pain relief, less opioid consumption, and less length of hospital stay. For function recovery, VAS score with activity and rates of nausea and vomiting for patients with THA, there were no obvious differences to be found. According to the published article written by Andersen et al^[20]^ in 2008, local infiltration was the systematic injection of analgesic agents into the ligaments, capsule, and other soft tissues during the surgery. Several advantages of this technique compared with traditional methods had been found, and the analgesia effects were limited only in the surgical area. Thereafter, its advantages like reduced pain, less analgesic consumption, and so on had been reported for many times. The results listed above revealed that infiltration of multimodal medicine in patients who had THA could lead to the reduction of analgesic like morphine or opioid consumption. Murphy et al^[8]^ reported less morphine use for patients with THA who received local infiltration of an anesthetic (bupivacaine), saline. In the most recent randomized, double-blind study, Villatte et al^[10]^ found the local infiltration technique provided effective pain relief for patients who received postoperative periarticular infiltration of THA with a local anesthetic (ropivacaine), adrenaline, but no effect on recovery and blood loss after surgery. Whereas Busch et al^[9]^ found that patients who had THA would need less postoperative analgesic requirements and also feel less pain when they tried to move their legs if they got the periarticular infiltration of an anesthetic including morphine, and ropivacaine, ketorolac, epinephrine.

However, as for the other outcomes, no statistical significance was found between the PDI group and control group in our analysis. But the meta-analysis by Jiang et al reported that periarticular multimodal drug injection was an efficient and safe adjunct for pain control, and it was superior to placebo for pain relief, opioid consumption, and short-term range of motion; they analyzed the impacts of different medicines used in the researches to the results and concluded that further study should be carried out to make different pesticide effects of different kinds of medicine in the total joint replacement surgery more clear.^[7]^ Undoubtedly, they did an excellent job regarding the analysis, but the method of incorporation of the medicine was not very clearly given, because we all know, medicine instilled to the articular cavity through a catheter or a needle is different from the way it is infiltrated into the soft tissue around the joint; we thought that they did not depart them clearly. Additionally, 1 important point we should notice is that the majority of patients included finally in the research by Jiang et al were Chinese and Korean, who are the people of the east of Asia; they all were the yellow race. But, the patients we included finally were all Europeans. This raised an important question that did the race of patients matter? Further studies could be carried out to make it more clear. Length of hospital stay was also an important issue for postoperative recovery. Most included studies^[9,11,13–16]^ reported that no statistical significance of the length of hospital stay was found in the analysis. Length of hospital stay may indicate the function recovery and also the speed of pain relief, which was a comprehensive character for the evaluation of the efficacy of local infiltration. The other 3 included studies^[8,10,12]^ did not report the length of hospital stay or did not give the detailed data about the outcomes.

Since we analyzed the local infiltration, local wound complications should be considered, but only 1 study^[9]^ reported that there were 3 patients who had a minor wound problem in the PDI group and 1 in the control group, but they suggested that it was caused by patients’ dressings and had nothing to do with the infiltration; other studies^[10,12,13,15,16]^ just reported that no significant difference was found between the 2 groups. About the system complication like nausea, vomiting, and urinary retention, most of the studies^[8–10,12–16]^ reported that no patient with the complication above in both groups was found. It may imply that the safety of the local medicine infiltration could be reasonably believed. There were some more outcomes in our analysis compared with the former ones, like the satisfaction of patients^[15]^ and the blood loss or the need for blood transfusion,^[10]^ but there was not enough patients and studies to compare with each other, and the outcomes of the 2 studies were relatively solo. Maybe more studies including the above outcomes should be searched and compared.

Our analysis had several limitations. First, compared with other studies, we only searched English articles, and unpublished trials were not included, which might lead to the publication bias. Second, the sample sizes were a little small; some outcomes like infection were only reported in large study groups. Third, included studies had some clinical heterogeneity. The most apparent limitation was different infiltration medicine in the protocol. Local infiltration drugs had various components, and the doses were different, either. The control group did not get any infiltration, which also could cause the inaccuracy. Finally, some outcomes like range of motion after surgery were not included ultimately because the data were not provided sufficiently, and it might lower the level of evidence.

But we have to admit that our analysis was special, because we only selected the studies which used the method of drug infiltration, that is, medicine was infiltrated into the soft tissue around the joint, rather than instilled into the articular cavity through a catheter or a needle; we departed the 2 methods clearly since we began to select studies. Additionally, the studies included finally were from 7 different countries, although it did not include China and Korea; may be it can give some reference to the countries of Europe. Only RCTs were identified, which was another strength of our study. Randomization and blindness were described adequately. Last but not the least, Preferred Reporting Items for Systematic Reviews and Meta-Analyses^[17]^ (PRISMA) guidelines and GRADE^[19]^ approach were applied in this study strictly.

### 4.1 Implications for practice and research

More research for the accurate method of the infiltration can be done in the future, like the site around the joint of medicine infiltration. Also, if the adjuvants in the infiltration can be uniformed in the research, the level of evidence can be pretty high.

## 5 Conclusions

In summary, intraoperative PDI can provide better pain relief, less opioid consumption, and less length of hospital, as compared with the placebo group. Also, it has no significant effect on VAS score with activity and complication rates of nausea and vomiting. Therefore, PDI may be recommended for the pain management after THA. However, due to the variations in the included studies, additional studies are needed to validate these conclusions.
